# Adverse birth outcomes and early-life infections after in utero exposure to corticosteroids for inflammatory bowel disease: a Danish nationwide cohort study

**DOI:** 10.1186/s12916-023-02817-7

**Published:** 2023-04-12

**Authors:** Line Riis Jølving, Jan Nielsen, Mette Louise Andersen, Sonia Friedman, Bente Mertz Nørgård

**Affiliations:** 1 Center for Clinical Epidemiology, Odense University Hospital, Kløvervænget 30, Entrance 216, 5000 Odense C, Denmark; 2 Research Unit of Clinical Epidemiology, Department of Clinical Research, University of Southern Denmark, Odense, Denmark; 3 Division of Gastroenterology, Hepatology, and Endoscopy, Department of Medicine, Brigham and Women’s Hospital, Boston, MA USA; 4 Harvard Medical School, Boston, USA

**Keywords:** Corticosteroids, In utero exposure, Birth outcomes, Congenital malformations, Preterm birth, Small for gestational age, Apgar score, Infant infections, Inflammatory bowel disease

## Abstract

**Background:**

Systemic corticosteroids are often used to treat inflammatory bowel disease (IBD) flares during pregnancy as maintenance of disease remission is crucial to optimize pregnancy outcomes. However, there is little data regarding the effect of in utero exposure to corticosteroids on the risk of adverse birth outcomes and early-life infections in the offspring.

**Methods:**

We used the Danish national registries to establish a nationwide cohort of all singleton live births in women with IBD from 1995 to 2015. Outcomes in children exposed in utero to corticosteroids were compared to those who were not exposed. In logistic and Cox proportional hazard regression models, we adjusted the outcomes (major congenital malformation, preterm birth, small for gestational age, low 5-min Apgar score, and infections) for confounders such as body mass index, smoking, comorbidity, and additional medical IBD treatment.

**Results:**

After in utero exposure to corticosteroids at any time between 30 days prior to conception through the first trimester (*n* = 707), the adjusted hazard ratio of major congenital malformation was 1.28 (95% CI: 0.82–2.00) compared to children born to women with IBD, but not exposed to corticosteroids in utero (*n* = 9371). After in utero exposure to corticosteroids at any time during pregnancy (*n* = 1336), the adjusted odds ratios for preterm birth, small for gestational age, and low 5-min Apgar score were 2.45 (95% CI: 1.91–3.13), 1.21 (95% CI: 0.76–1.90), and 0.91 (95% CI: 0.33–2.52), respectively. Finally, the adjusted hazard ratio of overall infections in the first year of life was 1.14 (95% CI: 0.94–1.39).

**Conclusions:**

This nationwide cohort study suggests that children of women with IBD exposed to corticosteroids in utero had an almost 2.5-fold increased risk of preterm birth. Use of corticosteroids is closely related to disease activity and we cannot adjust for the independent role of disease activity. It is however reassuring that the other examined birth and early-life outcomes were not statistically significantly increased.

**Supplementary Information:**

The online version contains supplementary material available at 10.1186/s12916-023-02817-7.

## Background

Inflammatory bowel disease (IBD) is prevalent in the fertile years and reproductive issues frequently arise [1]. Due to their immunosuppressive and anti-inflammatory effects, corticosteroids are often administered in pregnant women with IBD to treat flares because uncontrolled disease activity is regarded as more harmful to maternal and fetal health than high-dose steroids [2–4]. When systemic corticosteroid therapy is administrated in pregnancy, the corticosteroid crosses the placental barrier but might be in higher concentrations in the maternal circulation than in the developing fetus’ circulation, as it rapidly metabolizes to less active metabolites [1]. Despite the assumed limited fetal concentration, it is still debated whether the organogenesis is affected by maternal corticosteroid use during early pregnancy, where an increased risk of congenital malformations, with special attention to orofacial cleft defects, has been suggested in some studies [5–8]. Congenital malformations are always a matter of concern after drug exposure in pregnancy, but other adverse offspring outcomes are equally important. Preterm birth, impaired fetal growth, and low 5-min Apgar score are some of the most important outcomes that predict morbidity and mortality later in life [9, 10]. In utero exposure to corticosteroids among IBD patients has been associated with adverse birth outcomes such as preterm birth, small for gestational age, low birth weight, and neonatal infection in few recent studies [11–13]. It is hypothesized that corticosteroid exposure may influence immune system development and suppress the immune response in the neonate, leading to adverse outcomes such as neonatal infections. However, given that corticosteroids are reserved for patients with active inflammatory bowel disease, it is difficult to distinguish whether adverse birth outcomes arise due to active disease or corticosteroid exposure [3].

Based on nationwide population-based Danish register data, we aimed to examine if in utero exposure to corticosteroids in women with IBD is associated with an increased risk of major congenital malformation, preterm birth, small for gestational age, low 5-min Apgar score, and infections diagnosed in the first year of life, including an overall infection risk, and the risk of site-specific infection groups, compared to women with IBD not treated with corticosteroids during pregnancy.

## Methods

### Setting and study population

The tax-financed Danish healthcare system provides treatment and care of all residents in Denmark and this uniform organization of the healthcare system allows us to use a population-based study design on register data from central data sources which are routinely collected with complete follow-up [14, 15]. The Danish Civil Registration System includes information on dates of birth, migration, and death and holds a unique personal 10-digit key for linkage between registries [16]. We obtained data from the Danish Medical Birth Register [17], the Danish National Patient Register [18], and the Danish National Prescription Register [19]. All births in Denmark are registered in the Medical Birth Register established in 1973, and the register holds information regarding mother, child, and birth, e.g., age, body mass index (BMI), smoking status, parity, and gestational age, Apgar score, weight and length on the neonate. The Danish National Patient Register has collected information on all hospital discharges in Denmark since 1977, and since 1994 likewise on all outpatient visits. The information comprises diagnoses, surgeries and procedures performed, date of admission, and date of discharge, along with information on hospitals and departments. In 1994, the registration of diagnoses was changed from the Danish version of the International Classification of Diseases in the 8^th^ revision (ICD-8) to ICD-10 [18]. The Danish National Prescription Register has since 1995 collected data on all prescribed medications that have been redeemed in any Danish pharmacy to the citizens based on their civil registration number, and furthermore, the register provides information on the time and place of a redemption [19, 20]. All medications in the register are classified according to the Anatomical Therapeutic Chemical Classification (ATC) system. Note, throughout this paper, we refer to pregnant and birthing individuals as “female,” “women,” or “mothers” while acknowledging that not all individuals included in our study may choose this label.

### Study population

The study population comprises all singleton live births by women with IBD including ulcerative colitis (ICD-10: K51) and Crohn’s disease (ICD-10: K50), registered in the Danish National Birth Register from 1st January 1995 to 31st of December 2015. We had a 1-year follow-up on all children.

#### The exposed cohorts

For the children in the study population, we stratified the exposed cohort in two groups according to mothers’ use of corticosteroids during pregnancy. Cohort #1 was exposed within a period of 30 days prior to conception until the end of the first trimester, with at least one prescription of systemic corticosteroids (ATC: H02). This represents the period of organogenesis, and we used this cohort to examine major congenital malformations. Exposed cohort #2 was children exposed at any time during the entire pregnancy, i.e., at least one prescription of systemic corticosteroids from the date of conception to the date of birth [21]. This cohort was used to estimate the impact on adverse birth outcomes and early-life infections in the first year of life. We focused only on systemic corticosteroids, due to the reduced systemic bioavailability.

#### The unexposed cohort

From the study population, the unexposed cohort consisted of children born to women with IBD who were not treated with systemic corticosteroids within 30 days before conception or at any time during pregnancy.

### Outcome ascertainment

The designation congenital malformations included the first registered malformation diagnosed from the date of birth until the child turned one year, in the Danish National Patient Register, with the exclusion of minor birth defects according to the EUROCAT guidelines [22, 23]. Preterm birth was defined by birth before gestational week 37 + 0, and small for gestational age was estimated based on information on birth weight and gestation according to the Marsal et al. algorithm for small for gestational age [24]. Furthermore, we obtained the 5-min Apgar scores for the neonates, and scores below 7 were considered low [9]. All birth outcomes were derived from the Danish Medical Birth Register [17]. For the early-life infections, the outcomes were detected from the date of birth until the child turned one year, and was based on information from the Danish National Patient Register as ICD-10 codes, i.e., infections that were diagnosed at hospital admission. Mild infections that were diagnosed only by a general practitioner were thus not included in the outcome ascertainment. The type of infections was recorded according to site-specific groups, i.e., respiratory tract infections, infections of the gastrointestinal tract, urogenital infections, infections of the skin and subcutaneous tissue, bacteremia, and other infections. We also examined the overall risk of infection by estimating the risk of a first infection (regardless of type of infection). Please refer to Additional file 1: Table S1 for the included diagnostic ICD-10 codes, and corresponding infections, and for EUROCAT diagnoses of congenital malformation, Table S2.

### Data on covariates

The covariates were selected a priori from the different registries and applied to the statistical models when relevant. From the Medical Birth Register, we extracted data on maternal age at the time of birth (continuous), parity (0, 1 +), maternal smoking when entering the pregnancy (yes/no), sex of the child [25], calendar year of child birth distributed into the categories 1995–1999, 2000–2004, 2005–2009, and 2010–2015 and introduced in the models to adjust for a possible cohort effect, and maternal BMI (underweight [< 18.5 kg/m^2^], normal weight [18.5–24.9 kg/m^2^], overweight [25.0–29.9 kg/m^2^], and obese [25.0–29.9 kg/m^2^]). Information on BMI is available in the register from 2005. From the National Prescription Register [19], and the Danish National Patient Register, we extracted information on medications used to treat IBD, including biologic therapy from 2005 and onwards, azathioprine/mercaptopurine, 5-aminosalicylic acid derivatives (5-ASA) and methotrexate in a time period of 30 days prior to conception or during pregnancy. From the Danish National Patient Register [18], we obtained data on maternal comorbid diseases according to Charlson’s comorbidity index [26]. This index covers 19 major disease categories weighted according to their prognostic impact, and we computed the index based on diagnoses recorded during all previous hospitalizations since 1977, and in a two-level index defined as no comorbidity and some comorbidity (0, 1 +).

### Statistical analysis

The analytic unit of this study was births, and contingency tables were constructed for the main study variables according to the two exposed cohorts, and the unexposed cohort. The statistical model took the clustering of children born by the same mother into account. When examining the different dichotomous outcomes, i.e., risk of preterm birth, small for gestational age, and low 5-min Apgar score, we used univariate and multivariate logistic regression analyses by in utero corticosteroid exposure as crude and adjusted odds ratios (OR) with 95% confidence intervals (95% CI). In the logistic regression models, we adjusted for maternal age at child birth, calendar year of birth, maternal smoking, BMI, comorbidity, parity, the sex of the child, and medical treatment for IBD with biologic therapy, azathioprine/mercaptopurine, and 5-ASA. When we examined the time-to-event outcomes, i.e., major congenital malformation and the risk of infections within the first year of life, the follow-up of the children started on the date of birth and ended on the date of the first specified outcome of interest, emigration, death or the child’s first birthday, whichever came first. In order to analyze whether major congenital malformations or infections were different between the exposed and unexposed cohort, we used a Cox proportional hazard regression model estimating the hazard ratio (HR). First infection and each site-specific infection were visualized using Kaplan–Meier plots stratified for the IBD subtype, ulcerative colitis or Crohn’s disease. The distribution of the types of different major congenital malformations is presented in the Additional file 1: Table S3. The model assumptions were inspected graphically. In the Cox proportional hazard regression models, we adjusted for the same parameters as mentioned above. In general, for outcome categories with less than 5 child outcomes in the exposed cohort, we did not calculate the crude or adjusted HRs due to a lack of statistical precision.

All analyses were made using STATA Release 16 (StataCorp).

### Sensitivity/sub-analyses

In a first sensitivity analysis, we analyzed the confounding impact of maternal BMI as these data were only available for a restricted time period, and in a second sensitivity analysis, we excluded women with obstetrical diagnoses related to threatening preterm birth. Furthermore, in a sub-analysis, we stratified on a maternal underlying diagnosis of either ulcerative colitis or Crohn’s disease.

## Results

### Characteristics of the cohorts

In total, 6986 unique pregnant women with IBD were included in the study. Table 1 presents the basic study characteristics of the two exposed cohorts and the unexposed cohort, including characteristics of the mother and the childbirth. During the study period, a total of 707 children were born after early pregnancy exposure to corticosteroids, 1336 children were born after exposure at any time during pregnancy, and 9371 children were born to women with IBD who did not receive corticosteroids during pregnancy (unexposed). The maternal age distribution was identical in the three cohorts with a median age of 30 and 31 years at child birth (25–75 percentile: 28–34 years). The three cohorts had identical data on maternal BMI, smoking, comorbidity, and parity.

**Table 1:** Descriptive characteristics of women with inflammatory bowel disease (IBD), and their neonates exposed to corticosteroids during pregnancy, and unexposed from 1995 to 2015^*^

	**Exposed cohort #1** *N* = 707	**Exposed cohort #2** *N* = 1336	**Unexposed cohort** *N* = 9371
**Maternal age at child birth, median years (25–75 percentiles)**	31 (28–34)	30 (28–34)	31 (28–34)
**Maternal underlying disease, *N* (%)**			
Ulcerative colitis	477 (67.5)	963 (72.1)	5404 (57.7)
Crohn’s disease	228 (32.2)	367 (27.5)	3916 (41.8)
Both diagnosis	2 (0.3)	6 (0.4)	51 (0.5)
**Maternal disease duration, median years (25–75 percentiles)**	5 (2–10)	4 (1–8)	6 (3–11)
**Maternal BMI**^**1**^**, *N* (%)**			
< 18.5 (underweight)	25 (3.5)	50 (3.7)	313 (3.3)
18.5–24.99 (normal weight)	278 (39.3)	512 (38.3)	3904 (41.7)
25.00–29.99 (overweight)	81 (11.5)	160 (12.0)	1215 (13.0)
> 30.00 (obese)	41 (5.8)	94 (7.0)	710 (7.6)
Missing	282 (39.9)	520 (38.9)	3229 (34.5)
**Maternal smoking, *N* (%)**			
Non-smoker	564 (79.8)	1065 (79.7)	7389 (78.8)
Smoker	79 (11.2)	156 (11.7)	1444 (15.4)
Missing	64 (9.1)	115 (8.6)	538 (5.7)
**Maternal comorbidity, *N* (%)**			
No comorbidity	604 (85.4)	1169 (87.5)	8371 (89.3)
Some comorbidity	103 (14.6)	167 (12.5)	1000 (10.7)
Biologic therapy^**,***^			
No	684 (96.7)	1288 (96.4)	9154 (97.7)
Yes	23 (3.3)	48 (3.6)	217 (2.3)
Azathioprine/mercaptopurine^***^			
No	616 (87.1)	1192 (89.2)	8816 (94.1)
Yes	91 (12.9)	144 (10.8)	555 (5.9)
5-ASA^***^			
No	242 (34.2)	403 (30.2)	6731 (71.8)
Yes	465 (65.8)	933 (69.8)	2640 (28.2)
Methotrexate^***^			
No	707 (100.0)	1335 (99.9)	9370 (100.0)
Yes		1 (0.1)	1 (0.0)
**Calendar year of childbirth, *N* (%)**			
1995–1999	123 (17.4)	254 (19.0)	1513 (16.1)
2000–2004	184 (26.0)	303 (22.7)	1966 (21.0)
2005–2009	181 (25.6)	387 (29.0)	2426 (25.9)
2010–2015	219 (31.0)	392 (29.3)	3466 (37.0)
**Parity, *N* (%)**			
0	318 (45.0)	657 (49.2)	4442 (47.4)
1 +	389 (55.0)	679 (50.8)	4929 (52.6)
**Sex of the child, *N* (%)**			
Female	345 (48.8)	656 (49.1)	4609 (49.2)
Male	362 (51.2)	680 (50.9)	4762 (50.8)
**Maternal diagnoses, *N* (%)**			
O47 False labor	37 (5.2)	71 (5.3)	528 (5.6)
Z358B Previous preterm birth	20 (2.8)	32 (2.4)	227 (2.4)
P05-08 Intrauterin growth retardation			4 (0.0)
O14 Pre-eclampsia	26 (3.7)	40 (3.0)	290 (3.1)
O45 Abruptio placentae	5 (0.7)	9 (0.7)	56 (0.6)
O244 Gestational diabetes mellitus	19 (2.7)	39 (2.9)	211 (2.3)

^1^*BMI *Body mass index

^*^A total of 6986 women with inflammatory bowel disease

^**^In the register from 2005 and onwards. From 30 days prior to conception or during pregnancy

^***^Within 30 days prior to conception

Across the three cohorts, women had equivalent use of biologic therapy within 30 days prior to conception and during pregnancy. Slightly fewer women used azathioprine in the unexposed cohort; 5.9% vs. 12.9% and 10.8% in the two exposed cohorts. The same pattern was present regarding 5-ASA treatment during pregnancy with 28.2% in the unexposed cohort and 65.8% and 69.8% in the two exposed cohorts.

### Major congenital malformations

For major congenital malformations, the adjusted HR of the first diagnosed malformation was 1.28 (95% CI: 0.82–2.00) for children exposed to corticosteroids in early pregnancy, compared to children not exposed to corticosteroids (Table 2).

**Table 2:** The hazard ratio of congenital malformations in live born children exposed in utero to corticosteroids (#1), relative to children not exposed in utero to corticosteroids. Multivariable Cox proportional hazard regression model with crude and adjusted estimates with 95% confidence intervals (CI)

	**Live births after in utero exposure to corticosteroids #1** N=707		**Live births not exposed in utero to corticosteroids** **N=9371**		**Odds ratio (95% CI)**	
	*N* events	Years at risk	*N* events	Years at risk	Crude	Adjusted^a^
Congenital malformations	37	673.6	374	9021.8	1.32 (0.94–1.85)	1.28 (0.82–2.00)

^a^Adjusted for maternal comorbidity, maternal age at child birth, calendar year of child birth, maternal smoking status, parity, sex of the child, maternal BMI, maternal use of biologic therapy, azathioprines, or 5-ASA 30 days prior to conception or during pregnancy

### Preterm birth, small for gestational age, and low 5-min Apgar score

Table 3 presents the birth outcomes of preterm birth, small for gestational age and low 5-min Apgar score. The adjusted odds ratio of preterm birth after in utero exposure to corticosteroids at any time during pregnancy was 2.45 (95% CI: 1.91–3.13), and for small for gestational age and low 5-min Apgar score, the adjusted odds ratios were 0.91 (95% CI: 0.33–2.52) and 1.21 (95% CI: 0.76–1.90), respectively.

**Table 3:** Odds of adverse birth outcomes in live-born children exposed in utero to corticosteroids (#2), relative to children not exposed in utero to corticosteroids, multivariable logistic regression models with crude and adjusted estimates with 95% confidence intervals (CI)

	**Live births after in utero exposure to corticosteroids #2** *N* = 1336	**Live births not exposed in utero to corticosteroids** *N* = 9371	**Odds ratio (95% CI)**	
	*N* events (%)	*N* events (%)	Crude	Adjusted^a^
Preterm birth	186 (13.9)	641 (6.8)	2.20 (1.84–2.64)	2.45 (1.91–3.13)
Small for gestational age	45 (3.4)	264 (2.8)	1.20 (0.86–1.67)	1.21 (0.76–1.90)
Low 5-min Apgar score	16 (1.3)	50 (0.6)	2.27 (1.29–4.02)	0.91 (0.33–2.52)^b^

^a^Adjusted for maternal comorbidity, maternal age at child birth, calendar year of child birth, maternal smoking status, parity, sex of the child, maternal BMI, maternal use of biologic therapy, azathioprines, or 5-ASA 30 days prior to conception or during pregnancy

^b^Adjusted for maternal comorbidity, maternal age at child birth, calendar year of child birth, maternal smoking status, parity, sex of the child, preterm birth, maternal BMI, and maternal use of biologic therapy, azathioprines, or 5-ASA 30 days prior to conception or during pregnancy

### Infections in the first year of life

**Fig. 1:**
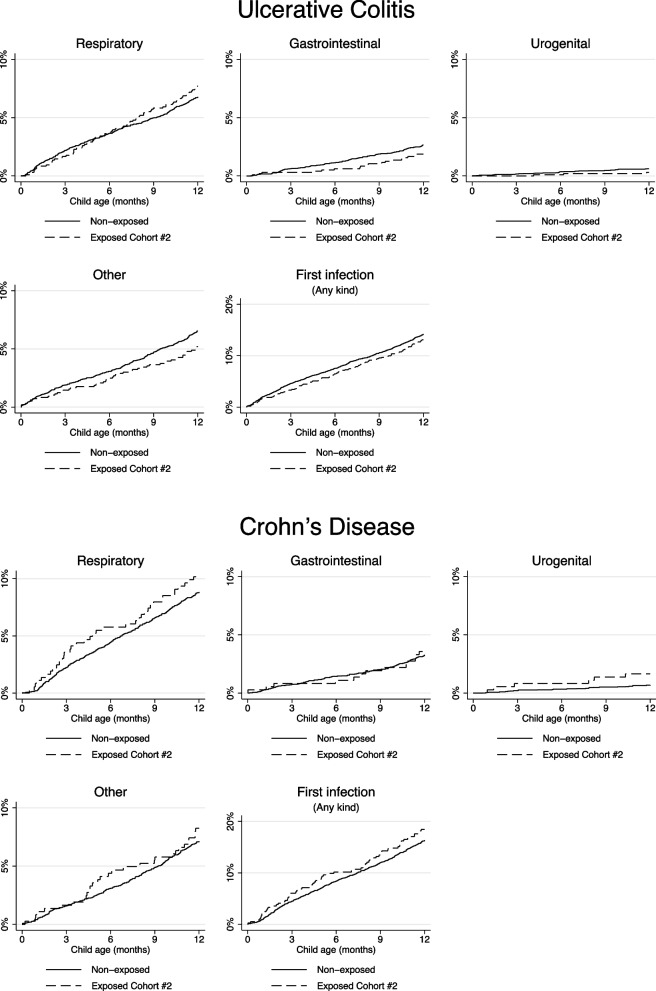
The cumulative proportion of infections from date of birth to one year of age according to the different site-specific infection categories, and first time infection, in children exposed in utero to corticosteroids, stratified on the maternal underlying disease, i.e., ulcerative colitis or Crohn’s disease

The crude and adjusted HRs for the site-specific infection categories in the offspring are presented in Table 4. In the group of first infection, covering all measured infections, the adjusted HR was 1.14 (95% CI: 0.94–1.39). There was no statistically significantly increased risk of the subtypes of infections, i.e., respiratory tract infections, infections of the gastrointestinal tract, urogenital infections, infections of the skin and subcutaneous tissue, bacteremia, and other infections after in utero exposure to corticosteroids compared to the unexposed children. We found no outcomes in the category of infections in the skin/subcutaneous tissue and bacteremia, and thus no sufficient analysis could be completed. For all other site-specific infections, the results are presented graphically as the cumulative proportions in Fig. 1 stratified on IBD subtype, i.e., ulcerative colitis and Crohn’s disease.

**Table 4:** Multivariable Cox proportional hazard regression model with crude and adjusted estimates and 95% confidence intervals (CI) and the hazard ratios for hospitalization with infection, and first time infection, in live born children exposed in utero to corticosteroids (#2), relative to children not exposed in utero to corticosteroids

	**Live births after in utero exposure to corticosteroids #2** *N* = 1336		**Live births not exposed in utero to corticosteroids** *N* = 9371		**Hazard ratios (95% CI)**	
Groups of child infections, 0–1 year	*N* events	Years at risk	*N* events	Years at risk	Crude	Adjusted^a^
First infection (any kind)	193	1234.2	1406	8614.2	0.96 (0.82–1.11)	1.14 (0.94–1.39)
Respiratory	111	1274.9	709	8972.8	1.10 (0.90–1.35)	1.18 (0.91–1.54)
Gastrointestinal	31	1318.3	273	9212.4	0.79 (0.55–1.15)	1.04 (0.66–1.63)
Urological/gynecological	9	1325.6	61	9302.8	1.04 (0.52–2.06)	1.69 (0.73–3.92)
Skin/subcutaneous tissue	-	1329.6	-	9333.3	-	-
Bacteremia	-	1328.0	17	9321.7	-	-
Other infections	80	1293.1	633	9033.1	0.88 (0.70–1.12)	1.23 (0.93–1.64)

^a^Adjusted for maternal comorbidity, maternal age at child birth, preterm birth, small for gestational age, calendar year of child birth, maternal smoking status, parity, sex of the child, maternal BMI, maternal use of biologic therapy, azathioprines, or 5-ASA 30 days prior to conception or during pregnancy

### Sensitivity/sub-analyses

In the sensitivity analyses on the confounding impact of maternal BMI, we did not detect any altered risk estimates of major congenital malformations, preterm birth, small for gestational age, low 5-min Apgar score, or infections, compared to the main analysis (data not shown). In the second sensitivity analysis excluding patients with obstetrical diagnoses, the risk estimates obtained from the main model did not alter (data not shown). Finally, in the sub-analysis stratifying the women according to their underlying IBD diagnosis of either ulcerative colitis or Crohn’s disease, we did not detect an impact of underlying maternal disease on the risk estimates. Data not shown.

## Discussion

Our results are based on all live-born children in women with IBD in Denmark from 1995 to 2015 (6986 individual women), and represent important and useful information for pregnant women with IBD, their partners, and clinicians. We found that neonates born to women with IBD who were treated with corticosteroids during pregnancy had a significantly increased risk of being born preterm. We found no statistically increased risk for the other examined outcomes, i.e., small for gestational age, low 5-min Apgar score, major congenital malformations, and hospital-diagnosed infections.

Although most women with IBD may conceive while their IBD is in remission, disease activity and flares can complicate up to 20% of all pregnancies in women with Crohn’s disease and 35% in women with ulcerative colitis [27]. Corticosteroids are frequently prescribed to treat IBD flares during pregnancy [3]. In a Danish study from 2016, Plauborg et al. found that 60% of women with Crohn’s disease and 51% of women with ulcerative colitis, had at least one prescription of corticosteroids during the period of one year before conception to one year post-partum [28]. Former studies have pointed towards the importance of disease activity itself, and active disease has been associated with an increased risk of adverse pregnancy and birth outcomes such as preterm birth [29–33]. In terms of measuring the effect of corticosteroids on birth and neonatal outcomes, it is difficult to separate the effects of maternal active IBD from the effects of corticosteroids. Active inflammation and use of corticosteroids to treat IBD flares are intertwined, and it is probably not possible to fully understand which factors may have contributed to a particularly adverse outcome. These challenges are not novel [12, 30, 34, 35]. A recent US study based on data from the multicenter prospective PIANO (Pregnancy in IBD Neonatal Outcomes) registry on a total of 432 children exposed in utero to corticosteroids[12] had a similar study question. The study examined the risk of congenital malformations, though not using the EUROCAT classification, and adverse birth outcomes such as preterm birth, small for gestational age, and infections occurring in the first year of life. The PIANO study limited the use of confounder adjustment and was not based on unselected nationwide data.

Regarding in utero exposure to corticosteroids and congenital malformations, first-trimester exposures to corticosteroids have been thought to confer an increased risk of orofacial clefts [5–7]. In a study from 2015, the authors found an association between Crohn’s disease and congenital malformations, with a relative risk of 1.85 (95% CI: 1.06–3.21), but no association with ulcerative colitis [34]. Yet these estimates were not adjusted for potential confounders such as maternal age and year of birth. Our data suggested no statistically significant increased risk with an adjusted HR of 1.28 (95% CI: 0.82–2.00) for major congenital malformation in offspring born to women with IBD treated with corticosteroids in early pregnancy. Furthermore, our stratified analysis on the IBD subtype did not alter these findings on major congenital malformations, and our findings are similar to the PIANO study that also showed no increased risk of congenital malformations 1.22 (95% CI: 0.80–1.87) [12].

Additionally, in women with Crohn’s disease, the exposure to topical or systemic corticosteroids has been examined according to various birth outcomes including preterm birth, congenital abnormalities, and low birth weight at term in 73 women with corticosteroids prescribed during pregnancy. None of the risk estimates provided were significant [11, 36]. Our study found a 2.5-fold risk of preterm birth in women using corticosteroids during pregnancy, which was also persistent in the stratified analysis by IBD subtype. Odufalu et al. also revealed a significantly increased risk of preterm birth with an adjusted odds ratio of 1.79 (95% CI: 1.18–2.73) in the PIANO study [12]. This was not confirmed in a recent study (*n* = 469) examining the risk of preterm birth by the time of exposure. Exposure in the first half of gestation provided an adjusted relative risk of 1.06 (95% CI: 0.59–1.89) and exposure with corticosteroids in the second half of gestation was not associated with an increased risk of preterm birth and adjusted hazard ratio 2.13 (95% CI: 0.99–4.59) [37]. We did not find an increased risk of small for gestational age after in utero exposure to corticosteroids compared to children not exposed. This is in accordance with previous literature [12]. Although such outcomes may seem related, they can vary regardless of the underlying mechanism. A child can be born at term and be small for gestational age but it can also be born preterm without being small for gestational age. There can be a link between the two outcomes but not necessarily. We were not able to address these aspects unambiguously in this register-based study [38].

An association between maternal IBD and low 5-min Apgar score has been examined in prior studies. Boyd et al. found an increased risk of a low 5-min Apgar score in neonates born at term to women with IBD, with a relative risk of 2.19 (95% CI: 1.03–4.66). This risk was increased further in neonates born to women with Crohn’s disease, relative risk 3.55 (95% CI: 1.45–8.67) [34]. However, the authors were not able to consider corticosteroid use in the statistical analysis due to a limited number of patients. These results are in contrast to a study from Sweden and Denmark showing no increased risk of low 5-min Apgar score in neonates born to women with Crohn’s disease, compared to women without IBD. This paper also did not consider steroid use during pregnancy [30]. Odufalu and colleagues found, that in utero exposure to corticosteroids in late pregnancy was associated with an increased risk of serious infections in the offspring at 12 months, odds ratio 2.9 (95% CI: 1.2–6.8) [12]. The study was based on self-reported data, and the identified increased risk was related to serious infections that required hospitalization and did not persist in the group of non-serious infections. In our study, we only included hospital-diagnosed infections. The studies are thus comparable regarding hospital admissions but the study from Odufalu et al. did not report on the type of infections using the ICD classification.

Corticosteroids may influence the development of the immune system but it is reassuring that we did not detect any statistically significant increased risk of infections in corticosteroid-exposed infants in the first 12 months after birth. Furthermore, we adjusted for preterm birth which per se is associated with an increased risk of early-life infections [39, 40].

This study has several strengths. Our nationwide cohort study examines the largest number of exposed infants to date with a unique opportunity for robust statistical risk estimates. We were also able to control for some of the most important confounders based on information from the national health registries which are highly valid, accurate, and complete [15]. We retrieved our outcomes independently from the exposure status preventing differential misclassification of the outcome measurement. In addition, we were able to conduct a complete 1-year follow-up on all children.

Our study also has limitations. In a study like this, it is not possible to separate the distinct effect of corticosteroids from an effect of disease activity but this applies to other studies in this area as well, as an indication for the use of corticosteroids is closely related to disease severity. These are well-known aspects and challenges in observational studies [12, 29, 35]. Whether disease flares may independently be associated with preterm birth, small for gestational age, low Apgar score, congenital malformations, and early-life infections directly, or through common causes such as smoking, maternal age, obesity, e.g., remains unanswered. Only live births were included in the analyses, as complete information on congenital malformations in stillbirths and late abortions was unavailable in the registries, which could have underestimated a potential impact on the outcomes. We cannot draw firm conclusions based on the small number of congenital malformations in our study, and we were not able to provide statistical analysis on the different subtypes of congenital malformations due to the lack of power (please refer to Additional file 1 Table S3). It is a limitation that we did not have access to data on the dosage and duration of the used medication in terms of a potential teratogenic impact, and when the outcome of interest is congenital malformations exposure with corticosteroids may have an impact on the embryogenesis. We have included available confounders but as in every observational study, we cannot rule out the presence of unknown or unmeasured residual confounding. We do not know the underlying mechanisms for the increased risk of preterm birth, or whether the finding is causal or susceptible for confounding. However, in general, the results for the birth and child outcomes were robust across the models adjusting for various confounders.

## Conclusions

In conclusion, corticosteroid use at any time during pregnancy in women with IBD was associated with a 2.5-fold increased risk of preterm birth. Corticosteroid use was not statistically significantly associated with any overall risk of major congenital malformations, being small for gestational age, having a low 5-min Apgar score, or hospital-diagnosed infections in the first year of life. Our study demonstrates and supports previous literature on the finding of an increased risk of preterm birth. Future studies in this area are warranted, especially studies that examine the underlying mechanisms for preterm birth but also other important outcomes such as neonatal hypoglycemia, neonatal intensive care unit admission, and extended postpartum hospitalization.

## Supplementary Information

**Additional file 1: Table S1.** All diagnoses of infections with related ICD-10 codes with the subdivision into site-specific groups. **Table S2.** List of ICD-10 codes used to define congenital malformations in accordance with the EUROCAT classification of malformations. **Table S3.** Distribution of congenital malformations in the exposed cohort #1 and the unexposed cohort.

## Data Availability

All data are stored and analyzed at a secure server at the Danish Health Data Authorities (Forskermaskinen at Sundhedsdatastyrelsen) using our institutional authorization. Any interested researcher can apply for access to health data through an application to the Research Service at the Danish Health Data Authority (forskerservice@sundhedsdata.dk). Access to data from the national health registries also requires approval from the Danish Data Protection Agency.

## Abbreviation

IBD: Inflammatory bowel disease

## References

[1] van der Woude CJ, Ardizzone S, Bengtson MB, Fiorino G, Fraser G, Katsanos K (2015). The second European evidenced-based consensus on reproduction and pregnancy in inflammatory bowel disease. J Crohns Colitis.

[2] Mitchell K, Kaul M, Clowse ME (2010). The management of rheumatic diseases in pregnancy. Scand J Rheumatol.

[3] Laube R, Paramsothy S, Leong RW (2021). Use of medications during pregnancy and breastfeeding for Crohn's disease and ulcerative colitis. Expert Opin Drug Saf.

[4] McGee DC (2002). Steroid use during pregnancy. J Perinat Neonatal Nurs.

[5] Park-Wyllie L, Mazzotta P, Pastuszak A, Moretti ME, Beique L, Hunnisett L (2000). Birth defects after maternal exposure to corticosteroids: prospective cohort study and meta-analysis of epidemiological studies. Teratology.

[6] Carmichael SL, Shaw GM, Ma C, Werler MM, Rasmussen SA, Lammer EJ (2007). Maternal corticosteroid use and orofacial clefts. Am J Obstet Gynecol..

[7] Gur C, Diav-Citrin O, Shechtman S, Arnon J, Ornoy A (2004). Pregnancy outcome after first trimester exposure to corticosteroids: a prospective controlled study. Reprod Toxicol.

[8] Fraser FC, Fainstat TD (1951). Production of congenital defects in the off-spring of pregnant mice treated with cortisone; progress report. Pediatrics.

[9] Gutbir Y, Wainstock T, Sheiner E, Segal I, Sergienko R, Landau D (2020). Low Apgar score in term newborns and long-term infectious morbidity: a population-based cohort study with up to 18 years of follow-up. Eur J Pediatr.

[10] Ehrenstein V, Pedersen L, Grijota M, Nielsen GL, Rothman KJ, Sorensen HT (2009). Association of Apgar score at five minutes with long-term neurologic disability and cognitive function in a prevalence study of Danish conscripts. BMC Pregnancy Childbirth.

[11] Norgard B, Pedersen L, Christensen LA, Sorensen HT (2007). Therapeutic drug use in women with Crohn's disease and birth outcomes: a Danish nationwide cohort study. Am J Gastroenterol.

[12] Odufalu FD, Long M, Lin K, Mahadevan U, Crohn's PIft, Colitis Foundation Clinical Research Alliance recruited patients for their respective centers for participant e. Exposure to corticosteroids in pregnancy is associated with adverse perinatal outcomes among infants of mothers with inflammatory bowel disease: results from the PIANO registry. Gut. 2021. 10.1136/gutjnl-2021-325317 34686575

[13] Broms G, Granath F, Stephansson O, Kieler H (2016). Preterm birth in women with inflammatory bowel disease - the association with disease activity and drug treatment. Scand J Gastroenterol.

[14] Epidemiology FL (2000). When an entire country is a cohort. Science.

[15] Schmidt M, Schmidt SAJ, Adelborg K, Sundboll J, Laugesen K, Ehrenstein V (2019). The Danish health care system and epidemiological research: from health care contacts to database records. Clin Epidemiol.

[16] Schmidt M, Pedersen L, Sorensen HT (2014). The Danish Civil Registration System as a tool in epidemiology. Eur J Epidemiol.

[17] Bliddal M, Broe A, Pottegard A, Olsen J, Langhoff-Roos J (2018). The Danish Medical Birth Register. Eur J Epidemiol.

[18] Schmidt M, Schmidt SA, Sandegaard JL, Ehrenstein V, Pedersen L, Sorensen HT (2015). The Danish National Patient Registry: a review of content, data quality, and research potential. Clin Epidemiol.

[19] Pottegard A, Schmidt SAJ, Wallach-Kildemoes H, Sorensen HT, Hallas J, Schmidt M (2017). Data Resource Profile: The Danish National Prescription Registry. Int J Epidemiol.

[20] Kildemoes HW, Sorensen HT, Hallas J (2011). The Danish National Prescription Registry. Scand J Public Health.

[21] Norgard BM (2011). Birth outcome in women with ulcerative colitis and Crohn's disease, and pharmacoepidemiological aspects of anti-inflammatory drug therapy. Dan Med Bull.

[22] Broe A, Damkier P, Pottegard A, Hallas J, Bliddal M (2020). Congenital Malformations in Denmark: Considerations for the Use of Danish Health Care Registries. Clin Epidemiol.

[23] anomalies Eesoc. EUROCAT Guide 1.4, Section 3.2 Minor Anomalies and other conditions for Exclusion 2005. Available from: https://eu-rd-platform.jrc.ec.europa.eu/system/files/public/Section%203.2__22_11_2021.pdf.

[24] Marsal K, Persson PH, Larsen T, Lilja H, Selbing A, Sultan B (1996). Intrauterine growth curves based on ultrasonically estimated foetal weights. Acta Paediatr.

[25] Kane S, Kisiel J, Shih L, Hanauer S (2004). HLA disparity determines disease activity through pregnancy in women with inflammatory bowel disease. Am J Gastroenterol.

[26] Charlson ME, Pompei P, Ales KL, MacKenzie CR (1987). A new method of classifying prognostic comorbidity in longitudinal studies: development and validation. J Chronic Dis.

[27] Pedersen N, Bortoli A, Duricova D, DI R, Panelli MR, Gisbert JP (2013). The course of inflammatory bowel disease during pregnancy and postpartum: a prospective European ECCO-EpiCom Study of 209 pregnant women. Aliment Pharmacol Ther.

[28] Plauborg AV, Hansen AV, Garne E (2016). Use of azathioprine and corticosteroids during pregnancy and birth outcome in women diagnosed with inflammatory bowel disease. Birth Defects Res A Clin Mol Teratol.

[29] Kammerlander H, Nielsen J, Kjeldsen J, Knudsen T, Friedman S, Norgard B (2017). The Effect of Disease Activity on Birth Outcomes in a Nationwide Cohort of Women with Moderate to Severe Inflammatory Bowel Disease. Inflamm Bowel Dis.

[30] Stephansson O, Larsson H, Pedersen L, Kieler H, Granath F, Ludvigsson JF (2010). Crohn's disease is a risk factor for preterm birth. Clin Gastroenterol Hepatol.

[31] Broms G, Granath F, Linder M, Stephansson O, Elmberg M, Kieler H (2014). Birth outcomes in women with inflammatory bowel disease: effects of disease activity and drug exposure. Inflamm Bowel Dis.

[32] Norgard B, Hundborg HH, Jacobsen BA, Nielsen GL, Fonager K (2007). Disease activity in pregnant women with Crohn's disease and birth outcomes: a regional Danish cohort study. Am J Gastroenterol.

[33] Bush MC, Patel S, Lapinski RH, Stone JL (2004). Perinatal outcomes in inflammatory bowel disease. J Matern Fetal Neonatal Med.

[34] Boyd HA, Basit S, Harpsoe MC, Wohlfahrt J, Jess T (2015). Inflammatory Bowel Disease and Risk of Adverse Pregnancy Outcomes. PLoS ONE.

[35] Bandoli G, Palmsten K, Forbess Smith CJ, Chambers CD (2017). A Review of Systemic Corticosteroid Use in Pregnancy and the Risk of Select Pregnancy and Birth Outcomes. Rheum Dis Clin North Am.

[36] Nørgård BM. Birth outcome in women with ulcerative colitis and Crohn’s disease, and pharmacoepidemiological aspects of antiinflammatory drug therapy [Doctoral dissertation]. University of Southern Denmark, Denmark: University of Southern Denmark, Denmark; 2011.

[37] Palmsten K, Bandoli G, Watkins J, Vazquez-Benitez G, Gilmer TP, Chambers CD (2021). Oral Corticosteroids and Risk of Preterm Birth in the California Medicaid Program. J Allergy Clin Immunol Pract.

[38] Blankenship SA, Brown KE, Simon LE, Stout MJ, Tuuli MG (2020). Antenatal corticosteroids in preterm small-for-gestational age infants: a systematic review and meta-analysis. Am J Obstet Gynecol MFM.

[39] Melville JM, Moss TJ (2013). The immune consequences of preterm birth. Front Neurosci.

[40] Steiner L, Diesner SC, Voitl P (2019). Risk of infection in the first year of life in preterm children: An Austrian observational study. PLoS ONE.

