# Supplementary material for: Adverse birth outcomes and early-life infections after in utero exposure to corticosteroids for inflammatory bowel disease: a Danish nationwide cohort study
Source: BMC Med. 2023 Apr 12;21:140. doi: 10.1186/s12916-023-02817-7 (PMC10091841; doi:10.1186/s12916-023-02817-7)
Supplement: Supplementary file 1 — Additional file 1: Table S1. All diagnoses of infections with related ICD-10 codes with the subdivision into site-specific groups. Table S2. List of ICD-10 codes used to define congenital malformations in accordance with the EUROCAT classification of malformations. Table S3. Distribution of congenital malformations in the exposed cohort #1 and the unexposed cohort. [file 12916_2023_2817_MOESM1_ESM.docx]

**ADDITIONAL FILES**

**Table S1.** All diagnoses of infections with related ICD-10 codes with the subdivision into site-specific groups

| **Infection groups** | **ICD-10** |
| --- | --- |
| **Respiratory tract infections** | |
| Tuberculosis  Parotitis | A15-A19  K11.2 |
| Nasopharyngitis | A36.1 |
| Sinuitis | J01 |
| Pharyngitis | J02 |
| Tonsillitis | A36.0; J03 |
| Laryngitis and tracheitis | A36.2; J04-J05; J37 |
| Acute upper respiratory infections of multiple and unspecified sites | A36.8-9; J06 |
| Abscessus peritonsillaris | J36 |
| Pneumonia | A48.1; B01.2; J10.0; J12-18; |
| Other acute lower respiratory infections | A37; A42.0; J20-22 |
| Abscessus pulmonis | J85 |
| Empyema pleurae | J86 |
| **Infections of the gastrointestinal tract** | |
| Intestinal infectious disease | A00-A09 (not A02.1) |
| Cholecystitis/Cholangitis | K81; K83.0 (not K83.0+ K83.0G) |
| Acute peritonitis | K65.0 |
| Hepatitis A, B, C | B15-B17 |
| Liver abscess | K75.0 |
| Abdominal actinomycosis | A42.1 |
| **Urogenital infections** | |
| Nephritis | N10 |
| Orchitis/epididimytis  Acute prostatitis | N45  N41.0 |
| Cystitis  Urethritis | N30.0  N34.1; N34.2 |
| Salpingitis and oophoritis | N70.0 |
| Endometritis | N71.0 |
| Cervicitis uteri | N72 |
| Syphilis | A50-A53 |
| Gonorrhea | A54 |
| Chlamydia | A55-A56 |
| **Infections of the skin and subcutaneous tissue** | |
| Erysipelas | A46 |
| Dermatophytosis and other superficial mycoses | B35-B36 |
| Cellulitis and abscess | L02-L03 |
| Acute lymphadenitis | L04 |
| Herpes virus | A60; B00-B02; H19.1 |
| Pilonidal cyst | L05 |
| Other local infections of skin and subcutaneous tissue  Rubella  Cocksackie | A36.3; L00; L01.0-1; L08; L30.3  H91.8A5; P35.0  A870A, B341A, J203 |
| **Bacterimia** | |
| Streptococcal | A40 |
| Other, including staph. aureus | A41 |
| Listeria | A32.7 |
| Salmonella | A02.1 |
| Candida | B37.7 |
| Meningococcal | A39.2 |
| **Other infections** | |
| Infection of the eye  Infection of the ear  Mastoiditis | B30; H00.0; H03.0-1; H06.1; H10.0; H10.5-8  H62-H66  H70 |
| Infections of the musculoskeletal system and connective tissue | M00-M01; M60.0; M86 |
| Infections in the nervous system | A39.0; A32.1; B00.3-4; B01.0; B02.1; A83; A87; B05.1; B06.0; G00-G02; G04-G06 |
| Certain bacterial disease | A15-A19; A2; A31-A32; A34-A35; A38.0; A42.2-9; A43-A44; A48.0; A48.2-9; A49 |
| Spirochaetal disease | A65-A69 |
| Rickettsiosis | A75-A79 |
| Viral infections | A8-A9; B05-B06; B08-B09; B25-B27; B33-B34 |
| Mycoses | B37-B39, B4; B59 |
| Protozoal diseases | B5-B7, B80-B83; A07.2 |
| Unspecified infectious diseases | B99.9 |

**Table S2.** List of ICD-10 codes used to define congenital malformations in accordance with the EUROCAT classification of malformations

| **Malformation group** | **Included ICD-10 codes**  **(incl. subgroups)** | **Excluded ICD-10 codes of minor malformations** |
| --- | --- | --- |
| All major malformations | Q  D181A D215 D821  P350 P351 P371 | Q078D Q078G Q101-Q105 Q135 Q170-Q175 Q179-Q182 Q184-Q187 Q189 Q211C Q246 Q254E Q261 Q314 Q318H Q320 Q322 Q331 Q357 Q381 Q382 Q385B Q400 Q401 Q430 Q444 Q458B Q501 Q502 Q505 Q523 Q525 Q527 Q53 Q544 Q552F Q552B Q610 Q627 Q633 Q653-Q656 Q658 Q659 Q661- Q669 Q670-Q675 Q678 Q680 Q682A Q683 Q684 Q685 Q740G Q752 Q753 Q760 Q764L Q765 Q766A-Q766C Q767C Q825 Q833 Q845 Q846 Q899 Q95 |
| Nervous system | Q00-Q07 | Q078D Q078G |
| Eye | Q10-Q15 | Q101-Q103 Q105 Q135 |
| Ear, face and neck | Q16-Q18 | Q170-Q175 Q179 Q180-Q182 Q184-Q187 Q189 |
| Heart Defects | Q20-Q26 | Q211C Q246 Q254E Q261 Q250 Q256 |
| Respiratory | Q300 Q32-Q34 | Q320 Q322 Q331 Q336 |
| Oro-facial clefts | Q35-Q37 | Q357 |
| Digestive system | Q38-Q45 Q790 | Q381 Q382 Q400 Q401 Q430 Q444 Q458B Q385B |
| Abdominal wall defects | Q792 Q793 Q795 |  |
| Urinary | Q60-Q64 Q794 | Q610 Q627 Q633 |
| Genital Organs | Q50-Q52 Q54-Q56 | Q501 Q502 Q505 Q523 Q525 Q527 Q552F Q544 Q552B |
| Limb | Q65-Q74 | Q653-Q656 Q658 Q659 Q661-Q669 Q670-Q678 Q680 Q683-Q685 Q682A Q740G |
|  |  |  |
| Chromosomal | Q90-Q93 Q96-Q99 | Q936 |
| Genetic syndrome, Skeletal dysplasia, Congenital skin disorder | D821 Q447B Q619A Q740B Q751 Q754 Q77 Q780 Q782 Q783 Q784 Q785 Q786 Q787 Q788 Q80 Q81 Q82 Q87 Q936 | Q870D Q870F Q870G Q870I Q872E Q872G |
| Teratogenic syndromes with malformations | Q86 P350 P351 P371 |  |
| Other anomalies | Q206 Q240 Q411 Q412 Q418 Q710 Q712 Q713 Q720 Q722 Q723 Q730 Q750 Q793 Q795 Q798S Q870G Q872G Q890 Q893 Q894 |  |

**Table S3.** Distribution of congenital malformations in the exposed cohort #1 and the unexposed cohort

| **Congenital malformations** | **Exposed cohort #1**  **n (%)** | **Unexposed cohort**  **n (%)** |
| --- | --- | --- |
| Nervous system | - | 27 (7.2) |
| Eye | - | 16 (4.3) |
| Ear, face and neck | - | - |
| Heart Defects | 12 (32.4) | 113 (30.2) |
| Respiratory | - | 6 (1.6) |
| Oro-facial clefts | - | 11 (2.9) |
| Digestive system | - | 26 (7.0) |
| Abdominal wall defects | - | - |
| Urinary | 6 (16.2) | 38 (10.2) |
| Genital Organs | - | 33 (8.8) |
| Limb | 12 (32.4) | 77 (20.6) |
| Chromosomal | - | 18 (4.8) |
| Genetic syndrome, skeletal dysplasia, Congenital skin disorder | - | 12 (3.2) |
| Teratogenic syndromes with malformations | - | - |
| Other anomalies | - | 12 (3.2) |

Each child could have one or more outcome with congenital malformation and the numbers does not sum up to table 2 in the main analysis.
